# Examining the Potential of Developing and Implementing Use of Adiponectin-Targeted Therapeutics for Metabolic and Cardiovascular Diseases

**DOI:** 10.3389/fendo.2019.00842

**Published:** 2019-12-11

**Authors:** Ying Liu, Vivian Vu, Gary Sweeney

**Affiliations:** ^1^Metabolic Disease Research Division, iCarbonX Co. Ltd., Shenzhen, China; ^2^Department of Biology, York University, Toronto, ON, Canada

**Keywords:** adiponectin, therapeutic, small molecule, peptide, precision medicine

## Abstract

Cardiometabolic diseases encompass those affecting the heart and vasculature as well as other metabolic problems, such as insulin resistance, diabetes, and non-alcoholic fatty liver disease. These diseases tend to have common risk factors, one of which is impaired adiponectin action. This may be due to reduced bioavailability of the hormone or resistance to its effects on target tissues. A strong negative correlation between adiponectin levels and cardiometabolic diseases has been well-documented and research shown that adiponectin has cardioprotective, insulin sensitizing and direct beneficial metabolic effects. Thus, therapeutic approaches to enhance adiponectin action are widely considered to be desirable. The complexity of adiponectin structure and function has so far made progress in this area less than ideal. In this article we will review the effects and mechanism of action of adiponectin on cardiometabolic tissues, identify scenarios where enhancing adiponectin action would be of clinical value and finally discuss approaches via which this can be achieved.

Adiponectin, one of the most abundant circulating hormones in healthy individuals, exerts many beneficial effects on cardiometabolic health. Adiponectin functions through multiple signaling pathways that stimulate beneficial effects on metabolism, inflammation, atherosclerosis, and cardioprotective responses. Circulating levels of adiponectin decline under conditions of metabolic stress, including obesity and metabolic syndrome, and are associated with decreased adiponectin signaling. Thus, strategies that enhance or restore adiponectin action are currently being investigated as therapeutic approaches in the treatment of cardiometabolic disease.

## Structural Features of Adiponectin

Circulating adiponectin is primarily derived from adipose tissue, with lower-level expression detected in several other peripheral tissues (1–4). Adiponectin monomers are oligomerized within adipocytes prior to secretion and circulate as low molecular weight (LMW) medium (MMW) and high molecular weight (HMW) forms. Extensive post-translational modifications play a critical role in adiponectin forming these oligomeric complexes. In particular, the disulfide bond formed via Cys39 (human)/Cys36 (mouse) and the hydroxylation, glycosylation occurred on lysine residues (lys68, lys71, lys80, lys104) are essential for adiponectin to form the hexameric and oligomeric complexes, respectively (5, 6). The full-length adiponectin (fAd) oligomers can be cleaved by neutrophil elastase to liberate the carboxy-terminal globular domain (gAd) which itself possesses physiological activity (7, 8).

## Adiponectin Receptors and Signaling

Adiponectin action is mediated primarily through adiponectin receptors 1 (AdipoR1) and 2 (AdipoR2), which are non-G-protein-coupled receptors containing seven transmembrane domains, but with an internal N-terminus and external C-terminus (9). Both adiponectin receptors are rather ubiquitously expressed although some variations do occur, such as more abundant AdipoR1 expression in skeletal muscle and higher AdipoR2 expression in liver (9, 10). *In vitro* experiments in C2C12 myocytes utilizing siRNA demonstrated that suppression of AdipoR1 reduced gAd binding while AdipoR2 suppression primarily reduced fAd binding, and their respective downstream signaling and functional effects (9). The functional roles of adiponectin receptors have been examined in transgenic or knockout mouse models of AdipoR overexpression created by different research groups. Yamauchi et al. reported undetectable levels of adiponectin specific binding and action in AdipoR 1 and 2 double-knockout mice leading to glucose intolerance and insulin resistance in these animals. Both AdipoR1-null and AdipoR2-null mice exhibited similar phenotypes with both strains showing increased adiposity and insulin resistance (11). A consistent phenotype of insulin resistance was observed in AdipoR1 deficient mice (12, 13), however studies in which AdipoR2 was deleted have reported opposing phenotypes in terms of glucose tolerance and susceptibility to diet-induced insulin resistance (11–14).

Adiponectin binding to AdipoRs initiates a cascade of downstream signaling through the interaction of AdipoR to intracellular adaptor proteins (15) with APPL1 (adaptor protein containing pleckstrin homology domain, phosphotyrosine binding domain, and leucine zipper motif 1) acting as the primary adaptor protein mediating the metabolic effects of adiponectin (16). Adiponectin stimulation results in the binding of APPL1 to the cytoplasmic domain of AdipoR1 and AdipoR2 via the phosphotyrosine binding (PTB) and coiled coil (CC) domain of APPL1 (17). Subsequent translocation of LKB1 to the cytosol as well as calcium release from the endoplasmic reticulum through phospholipase C activates calcium/calmodulin-dependent protein kinase (13, 18, 19). AMPK activation is the central mechanism whereby adiponectin stimulates metabolic effects (6, 7, 10, 13, 17, 18, 20–26), induces NO-dependent vasodilation, inhibits the production of reactive oxygen species (ROS), and modulates mTOR signaling. In addition to AMPK activation, several AMPK-independent pathways exists whereby adiponectin is able to regulate insulin sensitivity, inflammation, glucose uptake, and ceramidase activity (27).

## Physiological Effects of Adiponectin and Implications in Cardiometabolic Disease

The diverse physiological functions of adiponectin in metabolic and cardiovascular tissues has significant implications in health and disease states. Multiple studies have established primarily beneficial effects of adiponectin on the regulation of metabolism, immunity, inflammation, cardiac remodeling, vasculature control and cancer (16, 28–30). The anti-diabetic actions of adiponectin include insulin sensitizing and insulin mimetic actions in liver and skeletal muscle, as well as protection against beta cell destruction in the pancreas (31). In addition to this, increased glucose transport and GLUT4 translocation by adiponectin in skeletal muscle is regulated by AMPK or p38 MAPK activation (17). Adiponectin increases fatty acid oxidation through PPARα enhanced expression of target genes in the liver (20, 22, 23) or through increased mitochondria biogenesis in skeletal muscle (13).

The cardioprotective effects of adiponectin can be attributed in part to effects on cardiac metabolism, apoptosis, autophagy and hypertrophy (32). Additional cardioprotection is mediated by the anti-inflammatory, antioxidant, and vasorelexant properties of adiponectin as well as its ability to inhibit atherogenesis (31). Initial *in vivo* studies examining the effect of adiponectin on atherosclerosis demonstrated that adenovirus-mediated overexpression of adiponectin (33) and gAd treatment (23) in apolipoprotein (apo) E-deficient mice resulted in reduced atherosclerosis. Systematic review and meta-analysis of human clinical trials suggests an important role of adiponectin in the development of atherosclerosis, as hypoadiponectinemia was associated with early carotid artery atherosclerosis lesions in healthy and metabolic disease populations (34). It should be noted that this association was weak (34) and not consistent across all studies (35) but *in vitro* experiments as well as animals studies have reported data supporting the anti-atherogenic properties of adiponectin. Adiponectin inhibits multiple steps involved in the development of atherosclerotic lesions including the reduction of macrophage lipid accumulation, inhibition of macrophage to foam cell formation, suppression of pro-inflammatory cytokines release and lymphocyte migration, inhibition of leukocyte and endothelial cell interaction, and suppression of vascular smooth muscle proliferation through the inhibition of atherogenic growth factors (31, 36). In the early development of atherosclerosis, adiponectin has been demonstrated to inhibit monocyte-macrophage migration, thus reducing the attachment of monocytes to injured endothelial cells and the formation of macrophage foam cells (37, 38). In addition to this, adiponectin can downregulation scavenger receptor A (SR-A) and acyl-coenzyme A: cholesterol-acyltransferase 1 (ACAT1) expression, both of which are important regulators of macrophage lipid accumulation and foam cell formation (39, 40) Adiponectin can also inhibit foam cell formation by modulating lipid metabolism and cholesterol efflux in macrophages (41, 42) and altering the activity of enzymes (i.e., heptaic lipase, lipoprotein lipase) involved in the catabolism of lipoproteins (41). The effects of adiponectin on foam cell formation is mediated through an adiponectin-AdipoR1/2-APPL1 axis in macrophages (43, 44). Thus, adiponectin is a critical factor regulating the development of atherosclerosis which accounts for its beneficial effects on cardiometabolic diseases.

Dysregulation of cardiac energy metabolism is an important feature seen in early stages of heart failure. Cardiomyocyte contractile energy in the healthy heart under aerobic conditions is derived from fatty acids (~70%) and glucose (~30%) and this balance is perturbed under conditions of cardiac stress, such as under ischemic conditions (45). Our group (46–48) and others (49–51) have shown that adiponectin can regulate cardiac energy metabolism. Both globular and full-length adiponectin stimulation led to increased glucose uptake and metabolism (46) via the activation of AMPK, IRS1, and Akt1 in primary neonatal rat cardiomyocytes. The effect of adiponectin on cardiomyocyte glucose uptake is dependent on Rho/ROCK regulated actin cytoskeleton remodeling which colocalized APPL1 to actin filaments (48). Similar results were observed in primary adult rat cardiomyocytes with adiponectin significantly enhancing insulin stimulated Akt phosphorylation and glucose uptake (47). The effect of lipotoxicity on cardiac insulin signaling was examined in adiponectin knockout mice and a significant reduction in insulin stimulated Akt signaling was observed in high fat fed mice compared to chow fed group and adiponectin replenishment was able to reverse the defect in insulin signaling (52). Adiponectin treatment increased the expression of fatty acid transporter protein-1 and induced the translocation of CD36 to the outer cell membrane in primary neonatal and adult rat cardiomyocytes, respectively, resulting in increased fatty acid uptake (47, 48). Adiponectin, via AdipoR1-APPL1 signaling, regulates fatty acid β-oxidation in primary rat cardiomyocytes and isolated working heart through the activation of AMPK leading to acetyl coenzyme A carboxylase (ACC) phosphorylation and enhanced fatty acid oxidation (47, 48). These results suggest that adiponectin isoforms regulate cardiac metabolism and function leading to more efficient utilization of glucose and fatty acids.

Adiponectin attenuates cardiomyocyte apoptosis seen in heart failure through several different signaling pathways. Adiponectin treatment was protective against stress-induced apoptosis through Akt-dependent signaling (53) in HL-1 cardiomyocytes. In cultured H9c2 cells and adiponectin-null mice, globular adiponectin attenuated hypoxia/reoxygenation-induced apoptosis through the reduction of reactive oxygen species (54, 55). Adiponectin stimulation of ceramidase activity plays a vital role in cell survival through ceramide degradation and production of sphingosine 1-phosphate (S1P) both of which protected primary mice neonatal ventricular cardiomyocytes from palmate-induced apoptosis (56). Our group has recently demonstrated the importance of ceramidase activity in the cardioprotective effects against reactive oxygen species (ROS) and apoptosis. High fat diet (HFD) in adiponectin knockout mice increased myocardial total triglyceride, ceramides, and sphingosine-1-phosphate (S1P) compared to chow-fed animals with adiponectin replenishment resulting in significant reduction in S1P intracellular content. When these conditions were simulated in an *in vitro* model of lipotoxicity, palmitate treatment significantly increased S1P intracellular concentrations in H9c2 cells. Addition of a synthetic adiponectin receptor agonist, AdipoRon, to palmitate-treated H9c2 cells reduced intracellular concentrations while simultaneously increasing secretion of S1P which was consistent with attenuation of palmitate-induced ROS production and cell death. Thus, *in vivo* and *in vitro* results suggests that through the activation of cardiac ceramidase activity, adiponectin increases the conversion of ceramide to S1P which is secreted to exert autocrine/paracrine cardioprotective effects (52).

Regulation of cardiomyocyte autophagy is now recognized as an important mechanistic component of the cardioprotective effects of adiponectin. Examination of myocardial autophagy in an *in vivo* animal model of oxidative stress induced by chronic angiotensin II (Ang-II) treatment showed increased LC3II protein expression in the left ventricle which was significantly higher in adiponectin knockout vs. wild-type mice (57), this most likely resulting from a block of autophagic flux. H_2_O_2_ induced autophagic cell death in isolated adult rat ventricular myocytes through AMPK/ERK activation, mTOR inhibition, and increased expression of authophagic protein. This led to autophagasome accumulation and adiponectin pre-treatment ameliorated the effect of oxidative stress on autophagy (57). Cardiac autophagy is a dynamic process which is activated in response to stress but can become inhibited in chronic pathological conditions. In studies examining the effect of HFD on autophagy in wild-type and adiponectin knockout mice, a compensatory elevation in autophagy in response to HFD was lacking in Ad-KO mice (58). Aortic banding to induce pressure overload cardiac dysfunction resulted in activation of cardiac autophagy in wild-type mice but this change was deficient in adiponectin-null mice (59). Inhibited autophagy was also observed in adiponectin knockout mice following lipopolysaccharide challenge compared to wild-type mice which had upregulated LPS-induced autophagy through AMPK-mTOR-ULK1 dependent signaling (60). *In vitro* studies with H9c2 cells, revealed an important role of adiponectin in the stimulation of autophagy flux in cardiomyocytes at multiple steps within the autophagy pathway (59). In adiponectin deficient mice, the normal autophagic response of cardiomyocytes to stressors is inhibited and *in vitro* data suggests that adiponectin replenishment can correct this abnormal response. Collectively, *in vitro* and *in vivo* animal studies provide convincing evidence of multiple mechanisms in which adiponectin can enhance cell survival and metabolism through the regulation of autophagy.

Cardiac hypertrophy can be a normal physiological response which occurs secondary to pressure overload, however under specific circumstances hypertrophy can become pathological and lead to cardiac dysfunction (32). Adiponectin has been demonstrated to protect against pathological cardiac hypertrophy in both *in vitro* and *in vivo* experiments. Multiple studies have reported exaggerated pathological cardiac hypertrophy in adiponectin knockout mice under experiment models of pressure overload (61–63) with adiponectin replenishment resulting in reduced pathologic hypertrophy (62). Experiments in isolated neonatal rat ventricular myocytes treated with angiotensin II to induce hypertrophy illustrated that the primary anti-hypertrophic mechanism of adiponectin occurred through AdipoR1-APPL1-AMPK activation resulting in suppression of nuclear factor kappa-B-induced hypertrophic growth signaling (64–66). These studies suggest that adiponectin can protect against cardiac hypertrophy and lack of adiponectin can lead to development of more severe pathological hypertrophy. In summary, evidence to date illustrates that the cardioprotective effects of adiponectin are mediated through effects on multiple cell types (e.g., endothelial cells, vascular smooth muscle cells, cardiomyocytes, fibroblasts, macrophages) involved in cardiac metabolism, survival and remodeling.

## The Role of Adiponectin as a Biomarker

Adiponectin circulates at high concentrations in humans and rodents (67) and hypoadiponectinemia is associated with metabolic syndrome across different ethnic groups (34, 68–70). Adiponectin levels in humans are inversely related to BMI and fat mass with reduced adiponectin mRNA expression, HMW adiponectin secretion and total serum levels observed in obesity, insulin resistance, T2DM, CVD, and metabolic syndrome (34, 68, 71–73). Human studies have reported an inverse correlation between adiponectin levels and the risk of cardiovascular morbidity and mortality. Hypoadiponectinemia has been associated with early carotid artery atherosclerosis lesions in humans (34, 74), while higher serum adiponectin levels are correlated with favorable cardiovascular risk profiles in both males and females (75, 76). Higher adiponectin levels were also associated with a reduced risk of developing type 2 diabetes and the subsequent risk of cardiovascular events in a large population of healthy patients (77). Consistent with these cardioprotective effects are studies demonstrating an association between persistent hypoadiponectinemia following acute myocardial infarct with increased risk of future major adverse cardiovascular events (78, 79). In contrast, multiple studies have reported increased adiponectin levels in patients with advanced cardiovascular disease. In a prospective study of men and women without initial diagnosis ischemic heart disease or HF, higher adiponectin levels was directly correlated with increased risk of heart failure (80). The EXAMINE trial found a direct relationship between adverse cardiovascular outcomes and higher adiponectin levels in type 2 diabetic patients with recent acute coronary syndrome (81). In patients with chronic heart failure, high adiponectin levels were associated with an increased risk of mortality (82). Clinical studies in older populations demonstrated a positive association between adiponectin levels and mortality in patients with heart failure (83, 84). The paradoxical association of adiponectin levels in relation to cardiovascular disease was further illustrated by observations from the Copenhagen City Heart Study which reported a positive relationship between high adiponectin and decreased risk factors for CVD in patients without initial CVD disease. This same study also found a positive direct correlation between adiponectin levels and all-cause mortality and major adverse cardiovascular events (76).

This adiponectin paradox has been proposed to occur secondary to adiponectin resistance, a hypercatabolic state or in response to elevated natriuretic peptides which are found in advanced CVD. In particular, both human and animal studies have suggested that adiponectin resistance as a consequence of AdipoR1 downregulation may be present in severe CVD and thus hyperadiponectinemia is a compensatory mechanism (85, 86). The wasting theory proposes that the hypercatabolic state in severe heart failure leads to increased adiponectin which is consistent with the inverse relationship between adiponectin and fat mass (87). Supporting this theory are observations showing hyperadiponectinemia only in the presence of cachexia in heart failure patients (88). Cardiac natriuretic peptides, atrial natriuretic peptide (ANP) and brain natriuretic peptide (BNP), are important mediators in the crosstalk between heart and adipose tissue. This was demonstrated in transgenic mice models where whole body as well as adipose tissue specific deletion of the NP clearance receptors, resulting in enhanced ANP action, protected mice from diet induced obesity and insulin resistance (89). In human cultured adipocytes, ANP stimulated lipolysis (90) and promoted the “browning” of white adipose tissue through enhanced energy expenditure via upregulation of mitochondrial biogenesis, respiration and UCP1 expression (91). Unpublished data from our group suggest that defective cardiac autophagy leads to reduced ANP-mediated inter-organ crosstalk leading to impaired glycemic control. Increased natriuretic peptides release is an indicator of cardiac stress and is used as a clinical biomarker of heart failure. Both ANP and BNP were found to enhance the expression of adiponectin mRNA and secretion from primary human adipocytes (92). This observation was reproducible in human studies with infusion of ANP resulting in increased total and HMW adiponectin concentrations in healthy men (93) and patients with chronic heart failure (92). Clinical studies have also provided evidence for a positive, independent association between adiponectin levels and BNP in healthy subjects (94) and in men with ischemic heart disease (95). Concomitantly elevated proBNP was associated with elevated adiponectin levels observed in patients who developed heart failure in an 8.5 years follow-up prospective study of subjects without initial diagnosis of heart disease (80). Thus, both *ex vivo* and *in vivo* analyses have provided evidence supporting the idea that hyperadiponectinemia in advanced CVD can be driven by elevated natriuretic peptides.

Overall, the clinical value of adiponectin as a biomarker remains highly promising, especially for metabolic and cardiovascular diseases (96, 97). However, published findings suggest that clinical interpretation of circulating adiponectin levels must be done in the context of factors, such as age, gender, and severity or stage of CVD (98, 99).

## Adiponectin as a Biotarget

Research studies to date have proven the significant potential of adiponectin as a biotarget for the modulation of metabolic and cardiovascular disease. However, the exogenous administration of recombinant adiponectin has proven to be difficult due to the challenges associated with producing stable multimeric recombinant adiponectin isoforms. Further complicating the process of establishing a therapeutic dose is the high endogenous concentration and the relative short *in vivo* half-life of adiponectin (100, 101). The therapeutic success of adiponectin thus lies in approaches aimed at enhancing endogenous levels of expression as well as strategies to target adiponectin signaling and its downstream effector pathways.

## Current Knowledge and Future Approaches For How Adiponectin Can Be Targeted Therapeutically

### Increase Circulating or Local Levels

**Figure 1:**
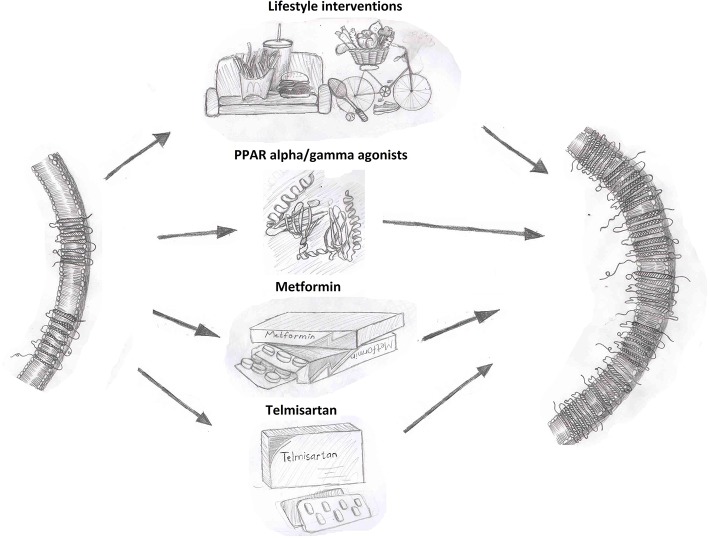
Interventions that enhance adiponectin receptor expression. Adiponectin receptor expression can be increased by lifestyle interventions, such as diet and exercise as well as through pharmacological therapies as shown.

As summarized in Figure 1, overcoming the reduction in circulating adiponectin levels observed in disease states, such as obesity and diabetes has potential to be beneficial. Lifestyle interventions are an effective strategy to increase circulating adiponectin levels. Interventions, such as exercise, caloric restriction, pharmacological drugs, or gastric bypass leading to weight loss have consistently shown a positive effect on adiponectin levels. Weight loss induced by a combination of lifestyle and pharmacological (phentermine/topiramate) interventions resulted in an increase in adiponectin levels in patients with metabolic syndrome (102, 103). Circulating adiponectin levels significantly increased following gastric bypass and sleeve gastrectomy in obese women and this was negatively correlated with body weight and waist circumference (104). Both gastric bypass and very low calorie diets led to improved adiponectin levels in obese type 2 diabetic subjects (105). Exercise training leading to weight loss in overweight, obese and diabetic subjects was associated with increased adiponectin levels (106–109). These findings support the idea that sustained weight reductions through lifestyle modifications can enhance adiponectin levels.

Thiazolidinediones (TZD), prescribed for the treatment of diabetes, are perhaps the most extensively characterized regulator of adiponectin expression. TZDs, such as pioglitazone and rosiglitazone increase adiponectin expression through the activation of proliferator-activated receptor gamma (PPARγ) (110, 111). Pioglitazone treatment for 16-weeks increased adiponectin levels in an obese population of Chinese subjects with diabetes and this correlated with improved insulin secretion and insulin sensitivity (112). Clinical studies in subjects across different ethnic, age and metabolic disease groups have consistently observed increased adiponectin levels with pioglitazone (113–115) or rosiglitazone (116) treatment. Importantly, TZDs up-regulate both total expression and HMW oligomers content in both *in vitro* as well as *in vivo* animal and human studies (3, 117, 118). However, the adverse side effects which have unfortunately blighted use of TZDs (119, 120) limit their utility as a go-to agent to increase adiponectin levels.

Specific dietary supplements have been recognized as regulators of adiponectin expression. Evidence from *in vitro* and *in vivo* studies suggests that vitamin E can upregulate adiponectin expression, also through the activation of PPARγ. Treatment with γ- and α-tocopherol, vitamers of vitamin E, enhanced adiponectin expression in 3T3-L1 adipocytes and mice (121) and was able to ameliorate the suppressive effects of TNF-α on adiponectin expression in 3T3-L1 adipocytes (122). Both fish oil (123) and omega-3 (124, 125) supplementation increased adiponectin levels in human studies. St. John's Wort (*Hypericum perforatum*) (126) and Groundsel Bush (*Baccharis halimifolia*) extract (127) induce 3T3-L1 adipocyte differentiation leading to enhanced adiponectin expression. Additional supplements that have been shown to increase circulating adiponectin in humans and animals include grape-seed extract (128–130) green tea extract (131, 132), walnuts (133, 134), α-linolenic acid (135), resveratrol (136–138), and *Radix Astragali* isolated compounds (astragaloside II and isoastragaloside I) (139).

Targeting the renin-angiotensin aldosterone system through angiotensin-converting enzyme inhibitor (ACEi) and angiotensin receptor blockers (ARBs) have consistently increased adiponectin levels in humans through PPARγ activated adiponectin gene transcription and enhanced adipogenesis (140). The ACEi inhibitor temocapril also significantly increased adiponectin expression in patients with essential hypertension (141). *Ex vivo* experiments with human omental (OM) preadipocytes from healthy women showed significantly higher adiponectin mRNA expression in adipocytes differentiated in the presence of ARBs vs. TZDs (140). Finally, it was suggested that enhanced adiponectin expression was specific to PPARγ ligand ARBs (e.g., irbesartan, telmisartan) via studies in 3T3-L1 adipocytes and *ex vivo* epididymal fat from Zucker *fa/fa* rats (142).

Incretins [e.g., glucagon-like peptide 1 (GLP-1), GLP-1 analogs (e.g. liraglutide)] and pharmacological agents used to increase incretin bioavailability [e.g., dipeptidyldipeptidase 4 (DPP4) inhibitors] upregulate adiponectin expression in 3T3-L1 adipocytes (143) as well as in clinical trials (144). Evidence from systematic review and meta-analysis of randomized controlled trials suggests that statins can increase adiponectin concentrations despite its negative effect on insulin sensitivity (145) and risk for developing diabetes mellitus (146). Empagliflozin, a diabetes pharmacological agent that works through the inhibition of sodium-glucose cotransporter-2, was been reported to increase adiponectin levels in mice through an unknown mechanism (147, 148). Together, these studies suggest that multiple pathways can regulate circulating adiponectin expression either through direct stimulation of gene expression or through mechanisms that enhance adipogenesis and insulin sensitivity.

### Alter Level (or Localization) of Adiponectin Receptors or Signaling Pathway Proteins

Regulation of AdipoR expression and downstream signaling effector proteins are clearly prime candidates which have been targeted to enhance adiponectin action and several compounds have been identified as possible regulators of adiponectin receptor expression. PPAR agonists have been reported to enhance AdipoR expression in various cell types. In epididymal white adipose tissue (WAT) of male KKAy mice, AdipoR1 and AdipoR2 expression was upregulated following treatment with the PPARα agonist Wy-14,643 (149). AdipoR2 but not AdipoR1 mRNA expression was increased by PPARα and PPARγ agonist in primary human and THP-1 macrophages (150). Rosiglitazone increased both AdipoR1 and AdipoR2 mRNA in isolated adult rat ventricular cardiomyocytes (51). In humans, pioglitazone treatment increased both AdipoR1 and AdipoR2 mRNA in muscle biopsies from type 2 diabetic subjects (151). Telmisartan, an ARB with selective PPARγ activity, was observed to correct the reduced ventricular cardiomyocytes AdipoR2 and aortic AdipoR1 in diabetic rat to comparable levels as control animals (152). Metformin, a first-line pharmacotherapy for treatment of diabetes, is a potent activator of AMPK with insulin sensitizing effects. Studies in ZDF rats demonstrated that metformin can upregulate AdipoR1 and AdipoR2 receptor expression in muscle and AdipoR1 in WAT (153). In addition to effects on adiponectin levels, exercise training in animals and humans is associated with enhanced AdipoR expression (109). Studies in obese and diabetic animal models consistently report upregulation of skeletal muscle AdipoR1 expression in response to different exercise programs (154, 155). In humans, AdipoR1 and AdipoR2 expression in skeletal muscle increased in response to endurance exercise programs (156, 157). Collectively, these findings further support the benefit of lifestyle interventions in enhancing adiponectin action.

### Receptor Agonists—What's Available and Future Developments

**Figure 2:**
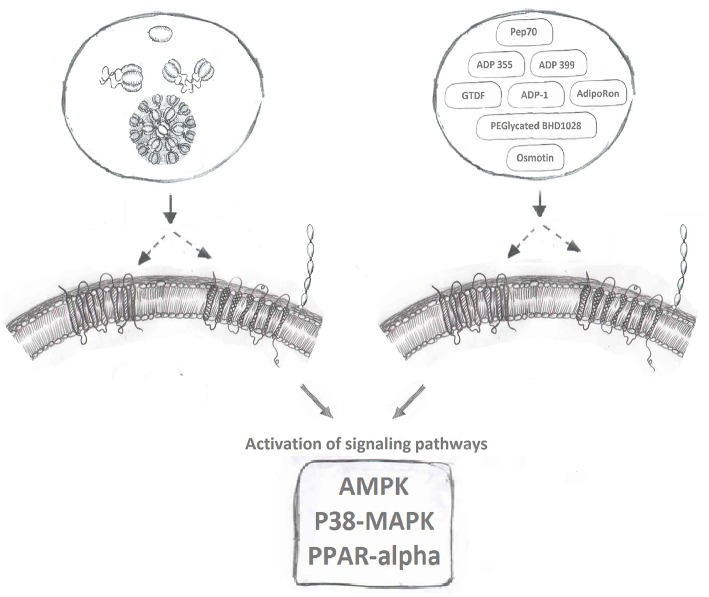
Adiponectin receptor agonists. Binding of full-length and globular adiponectin to adiponectin receptors with seven transmembrane spanning domains (left side) normally activates downstream signaling pathways, such as AMPK, p38MAPK, and PPAR-alpha. Multiple adiponectin receptor agonists have now been identified to also bind adiponectin receptors and induce these downstream signaling cascades.

AdipoR agonists (Figure 2) have been an intense focus of pharmaceutical drug development programs in metabolic and cardiovascular diseases (20, 158). Ligand binding receptors are of course recognized as the logical targets for activating/inhibiting that signaling pathway. Given the difficulties in producing biologically active adiponectin and optimizing the exact dosage and route of administration for this recombinant protein, designing agonists to activate adiponectin receptor-mediated downstream signaling is a highly desirable strategy to maximize adiponectin's beneficial effects. Several small molecules or short peptides have been discovered for this purpose since Kadowaki's group identified AdipoR1 and AdipoR2 (9).

## ADP355/ADP399

The C-terminal of adiponectin (residues 105–254) that forms gAd is well-established to induce potent biological effects in various studies (21, 159). By screening 66 small peptides overlapping each other by 10 amino acids and covering the entire sequence of the globular domain of adiponectin, Otvos et al. discovered that the peptide sequence between residues 149–166 retained biological activity similar to gAd (160). A patent was filed (US9,073,965) indicating the potential of developing this sequence into a receptor agonist. After additional pharmacological modifications and structure-function assays, a short peptide named *ADP355* (H-DAsn-Ile-Pro-Nva-Leu-Tyr-DSer-Phe-Ala-DSer-NH2), was formed acting as an active adiponectin receptor agonist. Follow up *in-vitro* activity assays including cell proliferation assay and western blot to monitor the activation of key signaling molecules (AMPK, AKT, STAT3, ERK1/2 etc.) in various cancer cell lines, revealed effects of *ADP355* in the concentration range 100 nM−10 uM comparable to or exceeding those of gAd (161). A bolus injection of 5–50 mg/kg *ADP355* displayed excellent stability and minimum toxicity *in-vivo*. Furthermore, a 1 mg/kg/day *ADP355* treatment via intraperitoneal (*i.p*.) injection for 28 days significantly inhibited the growth of human breast cancer xenograft in mouse (161). With the initial success, Otvos et al. continued to explore ways to pharmacologically enhance the agonist activity of *ADP355*. In considering adiponectin normally circulates in its multimeric forms, the second generation of the peptide, a linear branched dimer (*ADP399*) and an octapeptide (*ADP400*), was formed. The dimeric peptide *ADP399* exhibits almost 20-fold increased cellular activity in comparison to the monomeric peptide *ADP355*. However, surprisingly, at the similar concentration, *ADP400* acted as an antagonist rather than an agonist to AdipoRs (160).

## AdipoRON

AdipoRON, a synthetic small molecule that can be administered orally, is the most well-studied AdipoR agonist currently available. In 2013, via screening chemical library compounds provided by the Open Innovation Center for Drug Discovery in University of Tokyo, Kadowaki's group successfully identified AdipoRON as the most potent agonist for AdipoR's (162). During the screening assay, the phosphorylation of AMPK, the key targeted signaling molecule for adiponectin, was used as the readout for evaluating the activity of all chemical compounds in the screen. In C2C12 myotubes, comparing to the treatment of native adiponectin protein, AdipoRON induced a comparable and dose-dependent phosphorylation of AMPK within the concentration of 5–50 uM. AdipoRON was identified as agonist for both AdipoR1 and AdipoR2. An intravenous injection of AdipoRON, in the dose of 50 mg/kg, induced the phosphorylation of AMPK in both skeletal muscle and liver of wt mice, however, this phosphorylation was abolished in AdipoR1 and AdipoR2 double knockout mice (162). One key advantage of a small molecule receptor agonist in the pharma industry is oral administration. To test the therapeutic potential of AdipoRON in alleviating metabolic disorders, 50 mg/bodyweight of AdipoRON was given to diet induced obese mice via oral administration for 8 days. AdipoRON effectively improved insulin sensitivity and restored glucose homeostasis via the activation of AdipoR1-AMPK-PGC1α and AdipoR2-PPARα signaling pathways (162). AdipoRON treatment also mimicked adiponectin's established anti-diabetic effects (163) and ability to enhance cellular capacity for mitigating oxidative-stress (162, 164), enhancing lipid/glucose oxidation in mitochondria (162, 164), anti-inflammatory responses (162, 164–167), life-prolonging effect (162, 163), anti-cancer effects (168, 169), pro-cell survival and anti-apoptotic effect (170, 171), neuronal- (172, 173), reno- (174, 175), and cardio-/vascular-protective effects (165, 176–179). However, exciting AdipoRON research in animal models has not been translated to establishment of a drug for human use and the search continues for additional small molecule AdipoR agonists which have little or no toxicity.

## Osmotin

Osmotin, a plant protein, that was found to be structurally similar to adiponectin, can also induce the phosphorylation of AMPK in C2C12 myotubes (180). Interestingly, the adiponectin receptor, AdipoR1, is the mammalian homolog of the osmotin receptor, PHO36 (180). Based on these observations, it was proposed that osmotin could function as an agonist for AdipoR1 (181). Indeed, an osmotin peptide with nine residues (CTQGPCGPT) was synthesized and its molecular interaction with AdipoR1 was modeled *in silico*. Functional analyses revealed similar biological effects, evaluated as the induction of IL-6 production in synovial fibroblasts, of fAd (5 ug/ml) and osmotin peptide (5 ug/ml) (181, 182). Studies with *in-vivo, in-vitro* treatment of osmotin also revealed its adiponectin's memetic effect toward obesity, diabetes and related fatty liver, cardiovascular diseases (183–186). The activation of AdipoRs mediated downstream signaling pathways including AdipoRs/PPARα (185) and AdipoR1/PI3K/AKT (183) upon osmotin treatment, further confirmed the agonist activity of osmotin peptide to AdipoRs, in particularly to AdipoR1 (187). Interestingly, a group of Korean scientists led by Dr. Kim MO have conducted series of studies and revealed the neuroprotective effect of osmotin, in particular to Alzheimer disease. Firstly, they shown the preventive effect of osmotin on amyloid beta-induced synaptic deficits, Aβ accumulation, β-secretase expression and tau phosphorylation via reduced phosphorylation of PI3K, Akt, and GSK in mice (188). Secondly, they identified AdipoR1/TLR4/NFκB, AdipoR1/AMPK/SIRT1/SREBP2 signaling, and AdipoR1 interfered Nogo-receptor 1 (Ng1) signaling are three key pathways that responsible for osmotin diminished neuroinflammation and Aβ accumulation while improved neurodegenerative disease related, synaptic deficits, cognitive impairment, memory loss and long-term potentiation (189–192). Lastly, an osmotin preloaded magnetic nanoparticles demonstrated a novel drug delivery approach for the treatment of Alzeimer (193).

## Others

Additional AdipoR activating small molecules have also been studied as potential therapeutic agents. Via an *in silico* approach, peptide Pep70 was identified as a potential AdipoR1 agonist in protecting against fibrosis (194). PEGylated BHD1028 was discovered and formulated for the treatment of diabetes (195). Others identified that a short region of adiponectin protein N-terminus (6, 15–35) can act as agonist to AdipoRs and activate the downstream signaling pathways to promote cell viability and proliferation, thus maintaining pancreatic beta cell mass and preventing the development of diabetes (196). Another orally active AdipoR agonist named6-C-b-D-glucopyranosyl-(2S,3S)-(+)-5,7,39,49-tetrahydroxydihydroflavonol (GTDF) was identified and its biological effects characterized (197), followed by identification of a 13-amino acid residue segment located in the collagen domain of adiponectin (ADP-1) as another potent AdipoR1 agonist (198). The corresponding functional studies have revealed that GTDF can bind to both AdipoRs, with a preference for AdipoR1, and activate associated signaling pathways to improve metabolic health, including glucose uptake, lipid profile, beta cell survival, reduced steatohepatitis and the browning of adipose tissue (197, 198).

Many efforts have been invested in identifying and optimizing AdipoR agonists as a class of therapeutic drugs, however, none of them has reached the stage of being adopted in clinical practice yet. This may due to the interpersonal variants in endogenous adiponectin and adiponectin receptor expression levels and the complicated cross-reactive molecular networks formed around its signaling pathway. Besides, it is also important to realize that the development of adiponectin resistance in certain disease states, possibly due to the reduced expression or altered post-translational modification of adiponectin receptors or adaptor proteins, such as APPL1, could also significantly affect the effectiveness of AdipoR agonists (10, 199–201). However, with recent advances in high throughput and high content drug discovery technologies (202, 203) and the establishment of AdipoR crystal structure to facilitate rational drug design (204), it is possible to expect further refinement of small molecule AdipoR agonists for the treatment of metabolic and cardiovascular diseases.

## Future Considerations

### A Personalized Approach to Maximizing Effectiveness of Adiponectin Therapeutics

There clearly is huge potential for implementing use of adiponectin-based therapeutics, and when the time comes we must also make progress in stratifying ways to better deploy their use. Here, we list several possible directions that could be follow to maximize health impact of future advances in adiponectin-related drug discovery.

### Crosstalk With the Microbiome

As our understanding of the bio-effects of adiponectin has developed during the last decade, so too has knowledge of the important role that gut microbiota plays in metabolic health (205). Like adiponectin, targeting microbiota has also been suggested to have therapeutic value in treating metabolic syndrome. A large number of studies in rodents and humans have now validated the concept that insulin resistance condition could be improved upon receiving a more healthy donor's intestinal microbiota via a procedure called fecal microbiota transplantation (FMT) (206). Interestingly, it was found that expression of adiponectin can also be modulated by specific changes in gut microbiota composition. FMT, especially of those that are enriched in Lactobacillus NK6 colony (very similar to *Lactobacillus taiwanensis* strain BCRC 17755) can induce adiponectin expression from gut epithelial cells (207). Indeed, it has been proposed that activation of adiponectin signaling can also mediate the beneficial physiological effects of Lactobacillus (208). The important role of gut microbiota in mediating drug absorption and metabolism has now been recognized (209). Specifically, one study identified more than five phase-I metabolites and many possible glucuronic acid conjugated phase-II metabolites generated upon AdipoR agonist AdipoRON treatment (210). However, whether these metabolites are generated from microbe or host mediated drug metabolism, and whether there is any functional significance related to those metabolites are still under investigation. Thus, future studies focused on developing adiponectin-based therapies must include considering the dynamic interaction among gut microbiota, host metabolism and adiponectin based drug metabolism (211).

### Artificial Intelligence (AI) Assisted Drug Development and Use

The analysis of pharmacokinetics and pharmacodynamics on adiponectin based drug metabolism is often long and costly, yet artificial intelligence using machine learning algorithms are now able to assist this process. An example for AI assisted drug development is analysis of the hepatic toxicity effect of terbinafine, an oral antifungal agent (212). Nearly 20 years after the initial safety watch regarding its hepatic toxicity effect, a machine learning algorithm trained with large dataset of known metabolic pathways identified what no human could previously: the detailed 2-step metabolic processes that led to the breakdown of terbinafine into its toxic metabolite, TBF-A (213, 214). Using an AI assisted computational approach could help in forming a comprehensive understanding of metabolism for new adiponectin-mimetic small molecules and thus assist in making logical predictions of potential pharmacological considerations.

### Precision Medicine to Maximize Impact of Adiponectin-Based Therapeutics

Interpersonal variants in factors including the expression level of adiponectin and adiponectin receptors, the largely diverse life style (such as food composition, exercise frequency, sleep quality), the stage at which individuals are located along the course of disease progression, ultimately determine the individualized response to potential therapeutic interventions (215–218). The reciprocal interplay between microbiota and adiponectin action has also created personal variables in drug efficiency (208). All the above listed factors played important role on the yet not so successful translational trials on adiponectin based drug development. In order to design the most effective treatment plan according to all such variants, the concept of precision medicine is important. Several pioneering studies have illustrated the power of personalized profiling in understanding and capturing individualized characterization under the same clinical phenotype (219–221). For example, unique groups of small molecules were identified as being characteristic for each individual and that they differed from the group mean. These individualized datasets can serve as early predictive signatures for disease warning and allow specific preventive treatments to be planned accordingly in order to delay the occurrence of diseases. These pioneering studies provide an excellent guide in establishing a concept that will be applicable for future studies focused on adiponectin as a personalized therapeutic target.

**Figure 3:**
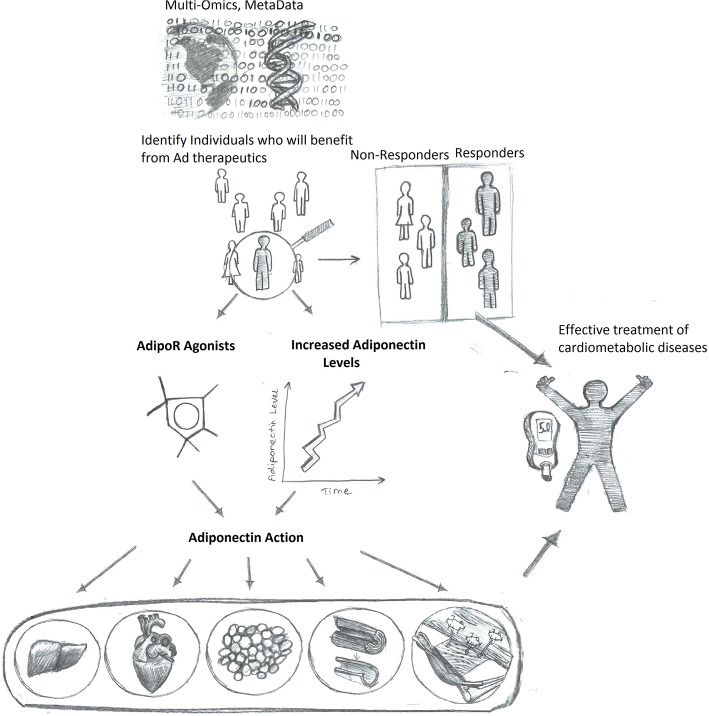
Enhanced adiponectin therapeutics via precision medicine upon personalized response prediction. Accumulation of big data will allow algorithm-based prediction of those individuals most likely to benefit from therapeutic agents to either increase adiponectin levels or directly mimic adiponectin action, thus resulting in more efficient and improved health care outcomes.

In conclusion, merging what we know about adiponectin in disease pathophysiology, and mechanisms of action with new understandings of how utilizing AI can personalize medicine and improve outcomes, it is hoped that adiponectin-based therapeutics can be tailored as the best-in-class approach for managing metabolic health in the future (Figure 3).

## Author Contributions

All authors listed have made a substantial, direct and intellectual contribution to the work, and approved it for publication.

### Conflict of Interest

YL was employed by iCarbonX. The remaining authors declare that the research was conducted in the absence of any commercial or financial relationships that could be construed as a potential conflict of interest.
